# *Apophysomyces variabilis*: draft genome sequence and comparison of predictive virulence determinants with other medically important *Mucorales*

**DOI:** 10.1186/s12864-017-4136-1

**Published:** 2017-09-18

**Authors:** Hariprasath Prakash, Shivaprakash Mandya Rudramurthy, Prasad S. Gandham, Anup Kumar Ghosh, Milner M. Kumar, Chandan Badapanda, Arunaloke Chakrabarti

**Affiliations:** 1 Department of Medical Microbiology, Postgraduate Institute of Medical Education and Research, Chandigarh, 160012 India; 2 Microbial Type Culture Collection and Gene Bank (MTCC), CSIR-Institute of Microbial Technology, Chandigarh, 160036 India; 3 Department of Biotechnology, Bhupat and Jyoti Mehta School of Biosciences, Indian Institute of Technology, Chennai, 600036 India; 4 Department of Biotechnology, OPJS University, Churu, 331303 Rajasthan India

**Keywords:** *Apophysomyces variabilis*, CotH proteins, Proteases, Transposons, Genome structure, Sequence analysis

## Abstract

**Background:**

*Apophysomyces* species are prevalent in tropical countries and *A. variabilis* is the second most frequent agent causing mucormycosis in India. Among *Apophysomyces* species, *A. elegans*, *A. trapeziformis* and *A. variabilis* are commonly incriminated in human infections. The genome sequences of *A. elegans* and *A. trapeziformis* are available in public database, but not *A. variabilis*. We, therefore, performed the whole genome sequence of *A. variabilis* to explore its genomic structure and possible genes determining the virulence of the organism.

**Results:**

The whole genome of *A. variabilis* NCCPF 102052 was sequenced and the genomic structure of *A. variabilis* was compared with already available genome structures of *A. elegans, A. trapeziformis* and other medically important *Mucorales*. The total size of genome assembly of *A. variabilis* was 39.38 Mb with 12,764 protein-coding genes. The transposable elements (TEs) were low in *Apophysomyces* genome and the retrotransposon Ty3-gypsy was the common TE. Phylogenetically, *Apophysomyces* species were grouped closely with *Phycomyces blakesleeanus*. OrthoMCL analysis revealed 3025 orthologues proteins, which were common in those three pathogenic *Apophysomyces* species. Expansion of multiple gene families/duplication was observed in *Apophysomyces* genomes. Approximately 6% of *Apophysomyces* genes were predicted to be associated with virulence on PHIbase analysis. The virulence determinants included the protein families of CotH proteins (invasins), proteases, iron utilisation pathways, siderophores and signal transduction pathways. Serine proteases were the major group of proteases found in all *Apophysomyces* genomes. The carbohydrate active enzymes (CAZymes) constitute the majority of the secretory proteins.

**Conclusion:**

The present study is the maiden attempt to sequence and analyze the genomic structure of *A. variabilis*. Together with available genome sequence of *A. elegans* and *A. trapeziformis,* the study helped to indicate the possible virulence determinants of pathogenic *Apophysomyces* species. The presence of unique CAZymes in cell wall might be exploited in future for antifungal drug development.

**Electronic supplementary material:**

The online version of this article (10.1186/s12864-017-4136-1) contains supplementary material, which is available to authorized users.

## Background

*Mucoromycotina*, a subdivision of Kingdom Fungi is currently placed in *incertae sedis* and comprises three orders namely *Mucorales*, *Endogonales* and *Mortieriellales* [1]. In the order *Mucorales* 14 families, 56 genera and around 225 species have been identified [2, 3]. The traditional taxonomy of mucoralean fungi was largely based on morphological features, sexual reproduction and ecological characters. The current taxonomical position of fungal species under the order *Mucorales* is evolving with the use of molecular techniques. Recently, Spatafora et al. proposed a phylum level phylogenetic classification of mucoralean fungi comprising of two phyla (*Mucoromycota* and *Zoopagomycota*), six subphyla, four classes, and 16 orders [4]. Of the known 225 species under *Mucorales*, approximately 20 species are known to cause human infections.

*Rhizopus arrhizus* is the predominant (~60%) agent causing mucormycosis worldwide [5, 6]. *Lichtheimia* and *Mucor* species are the next group of fungi isolated from mucormycosis cases in western world [7, 8]. In contrast, *Apophysomyces* species ranks second after *R. arrhizus* in India; around 60% of world mucormycosis cases due to *Apophysomyces* species are reported from this country [9–11]. Misra et al. isolated this fungus from the environment of a mango orchard of North India in 1979 for the first time [12]. Subsequently the fungus was reported from the soils with low nitrogen content in India [13]. *A. elegans* was considered the only species under the genus *Apophysomyces* causing human infection. However, the molecular phylogenetic studies in recent years revealed *Apophysomyces* genus encompasses 5 species: *A. elegans*, *A. mexicanus, A. ossiformis, A. trapeziformis* and *A. variabilis*; *A. elegans, A. trapeziformis,* and *A. variabilis* are common human pathogenic species [14, 15]. The majority of human infections are due to *A. variabilis* [16]. Though *Apophysomyces* species generally produce cutaneous and subcutaneous infection following trauma, occasional deep seated infections involving lung, paranasal sinuses and kidney have also been reported [9, 11, 17]. *A. variabilis* is the major pathogen in India and occasional cases are due to *A. elegans* [10]. *A. trapeziformis* has been reported from necrotising fasciitis cases among tornado victims from USA [18, 19]. The study on pathogenesis of mucormycosis draws attention of clinicians and scientists, as *Mucorales* are known to cause severe infection and high mortality. Recently, using transcriptome and genomic approach, various pathogenic determinants have been identified in *Mucorales* [20]. However, the genome of *A. variabilis* had not been sequenced and pathogenic determinants of *Apophysomyces* species were not clearly delineated. The present study reports the draft genome sequence of *A. variabilis* for the first time and compared the same with already available genomes of *A. elegans* and *A. trapeziformis* to identify the possible virulence determinants in pathogenic *Apophysomyces* species.

## Results

### Genome features and organisation

The whole genome shot-gun high quality reads of *A. variabilis* (NCCPF 102052) isolate were generated using pair-end and mate-pair libraries on Illumina platform. *A. trapeziformis* (B9324) and *A. elegans* (B7760) genomes were downloaded from NCBI genome database for the comparative analysis. The genome assembly of *A. variabilis* consisted of 1155 contigs and 431 scaffolds with N_50_ scaffold length of 647.5 kilobases (Kb) and total genome size of 39.38 Mb. Whereas, the genome sizes of *A. elegans* and *A. trapeziformis* were 38.46 Mb and 35.84 Mb respectively. The average G + C content of the three *Apophysomyces* genomes ranged from 41.7% to 41.9%. The genome annotation of the three *Apophysomyces* species was performed using AUGUSTUS, which predicted >12,000 protein coding genes with an average length ranging from 1969 to 2018 bp (Table 1). The protein coding genes had multiple exons, with an average of seven exons per gene and exon length of ~255 bp (Table 1). The genomes of *Apophysomyces* species had six introns per gene with an average length of ~77 bp.

**Table 1:** Salient features of genome assembly of three *Apophysomyces* species

Assembly Statistics	*A. variabilis*	*A. elegans*	*A. trapeziformis*
Number of scaffolds	431	1528	1400
Number of contigs	1155	1528	1400
Total size of scaffolds (bp)	39,387,571	38,467,560	35,840,759
Scaffold N50 (bp)	647,494	101,064	141,065
Average Scaffold size (bp)	91,386	25,175	25,601
Max. scaffold size (bp)	3,993,377	469,772	572,943
Min. scaffold size (bp)	500	301	205
GC Content (%)	41.65	41.69	41.90
Coding sequences (CDS)			
Number of CDS	12,764	12,829	12,108
Total size of CDS (bp)	25,752,686	25,262,110	24,132,511
CDS N50 (bp)	3141	3018	3067
Average CDS size (bp)	2018	1969	1993
Max. CDS size (bp)	34,923	35,019	35,013
Min. CDS size (bp)	57	44	43
GC Content (%)	45.49	45.54	45.69
Exons (bp)	97,463	98,425	94,508
Average exon size (bases)	263	255	254
Exons/gene	7.64	7.67	7.81
Number of tRNA genes	197	174	197
Non coding sequences			
Introns (bp)	79,821	80,964	78,014
Introns/gene	6.25	6.31	6.44
Average intron length (bp)	77	76	76

*bp*: base pairs, *CDS*: coding DNA sequences

### Protein coding gene prediction and annotation

**Fig. 1:**
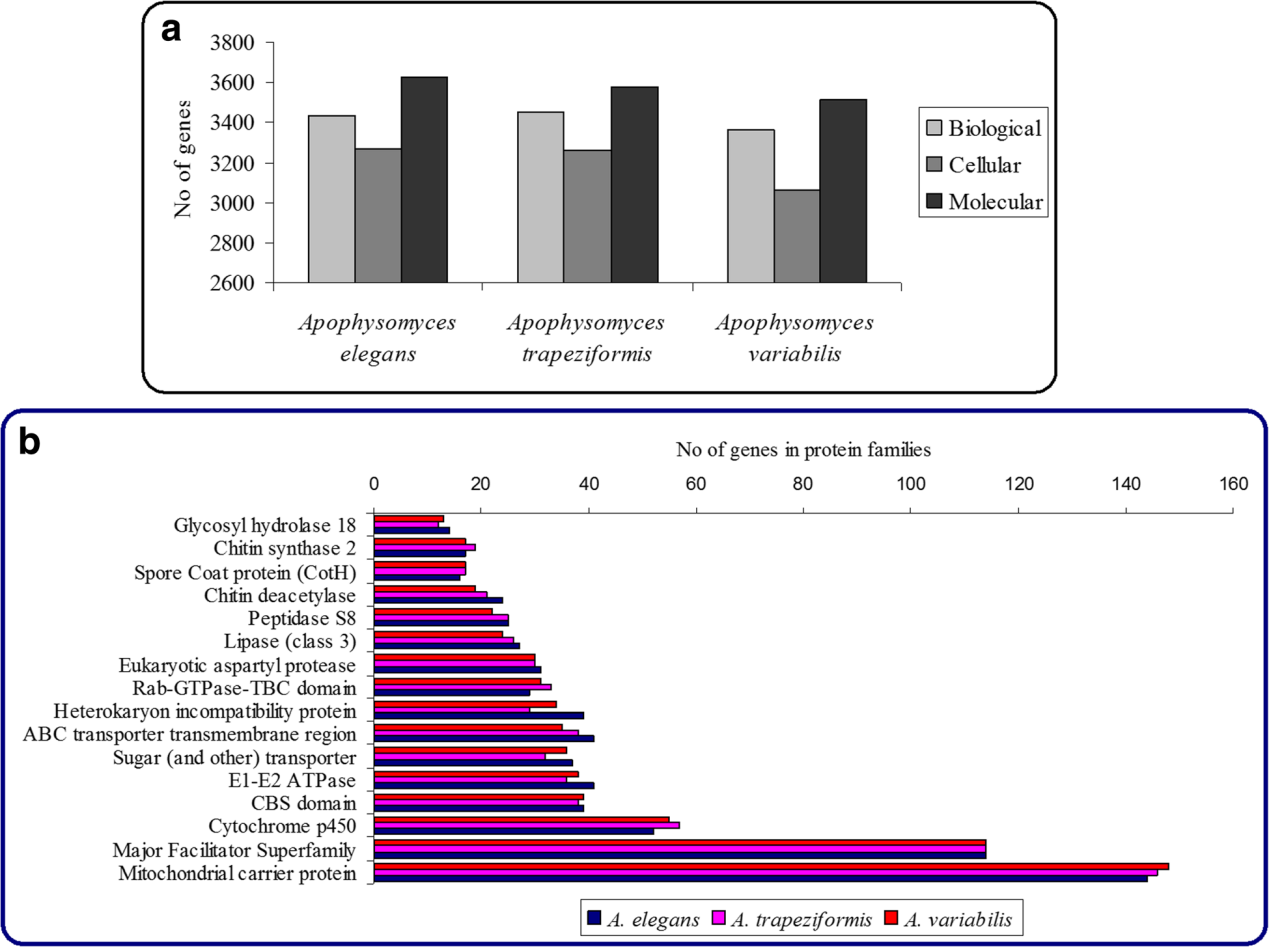
Gene ontology and major protein family classification in *Apophysomyces* genomes. **a**) Functional classification of proteins by Gene Ontology in three *Apophysomyces* genomes, **b**) major protein families annotated using PFAM in *Apophysomyces* genomes

Protein coding genes were functionally annotated using BLASTx (e-value less than 10^-5) from non-redundant protein database of NCBI. Approximately 88% of the genes in the genome were functionally annotated. The annotated genes were assigned to different Gene Ontology (GO) domains comprising of biological, cellular and molecular functions (Fig. 1a, Additional file 1: Figure S1). The PFAM annotation predicted 1877 predicted proteins for *A. variabilis*, 1875 for *A. trapeziformis* and 1884 for *A. elegans* (Fig. 1b). The BLASTx annotation of the predicted genes in *Apophysomyces* genomes revealed maximum hits with *Lichtheimia ramosa* (formerly *Absidia idahoensis*), followed by *Lichtheimia corymbifera* and *Rhizopus microsporus*.

### Transposable elements (TEs)

*Apophysomyces* genome contained low number of transposable elements. The proportion of TEs to the whole genome of *A. variabilis*, *A. elegans* and *A. trapeziformis* were 5.1%, 2.8%, 1.63% respectively. The retrotransposon Ty3-gypsy was the most abundant TE found in all three *Apophysomyces* genomes (Table 2). The low TE content in *Apophysomyces* species might be attributed to the expansion of RNA interference mediated genes and heterokaryon incompatibility (HET) proteins in their genomes.

**Table 2:** Transposons in genomes of three *Apophysomyces* species

Transposons	*A. variabilis*		*A. elegans*		*A. trapeziformis*	
	No of base pairs	% in genome	No of base pairs	% in genome	No of base pairs	% in genome
Class I (retro transposons)						
Ty3-gypsy	695,311	1.77	268,349	0.7	190,906	0.53
LINE	134,077	0.34	115,891	0.3	70,527	0.21
Ty1-copia	120,547	0.31	49,251	0.13	24,434	0.07
LTR-Roo	114,577	0.29	34,425	0.09	39,791	0.11
Total bases of retro transposons	1,064,512	2.7	467,916	1.2	325,658	0.91
Class II (DNA transposons)						
hAT element	291,667	0.74	165,177	0.43	30,059	0.08
Ant1	151,667	0.39	158,969	0.41	57,623	0.16
Mariner	127,820	0.33	137,342	0.36	54,268	0.15
MuDR	123,763	0.31	17,140	0.04	14,817	0.04
Helitron	103,738	0.26	67,336	0.18	52,081	0.15
CACTA elements	70,122	0.18	25,312	0.07	25,933	0.07
ISC1316	48,806	0.12	12,292	0.03	18,902	0.05
DDE transposon	30,481	0.08	29,761	0.08	3383	0.01
Crypton	–		1037	0.002	–	
P element	–		161	0.0004	–	
Total bases of DNA transposons	948,064	2.4	614,527	1.6	257,066	0.72
Total number of transposon bases in genomes	2,012,576	5.1	1,082,443	2.8	582,724	1.63

### Non-coding RNA

Annotation of tRNAs was performed using infernal tool. Comparison against RFAM database revealed 197 tRNAs in *A. variabilis* and *A. trapeziformis* and 174 tRNAs in *A. elegans* (Table 1). *Apophysomyces* genomes contained single copy of nuclear ribonuclease P (*RNase P*), a ribozyme that cleaves the 5′ regions of precursor tRNAs to generate mature 5′ ends of tRNA. Like all eukaryotes, the genome of *Apophysomyces* species contained all five (U1, U2, U4, U5 and U6) small nuclear ribonucleo-proteins (snRNA) essential for formation of major spliceosomes. Few components of minor spliceosome (U1, U6atac) were also present in the assembly. Other essential components like U4atac and U12 snRNA were not present. *A. variabilis* and *A. trapeziformis* contained group I introns, a self splicing ribozymes. The histone 3′ UTR stem-loop was present only in *A. variabilis* genome and it plays a major role in transport of the histone mRNAs from nucleus to cytoplasm (Additional file 2: Table S1).

### Orthologs and paralog prediction

**Fig. 2:**
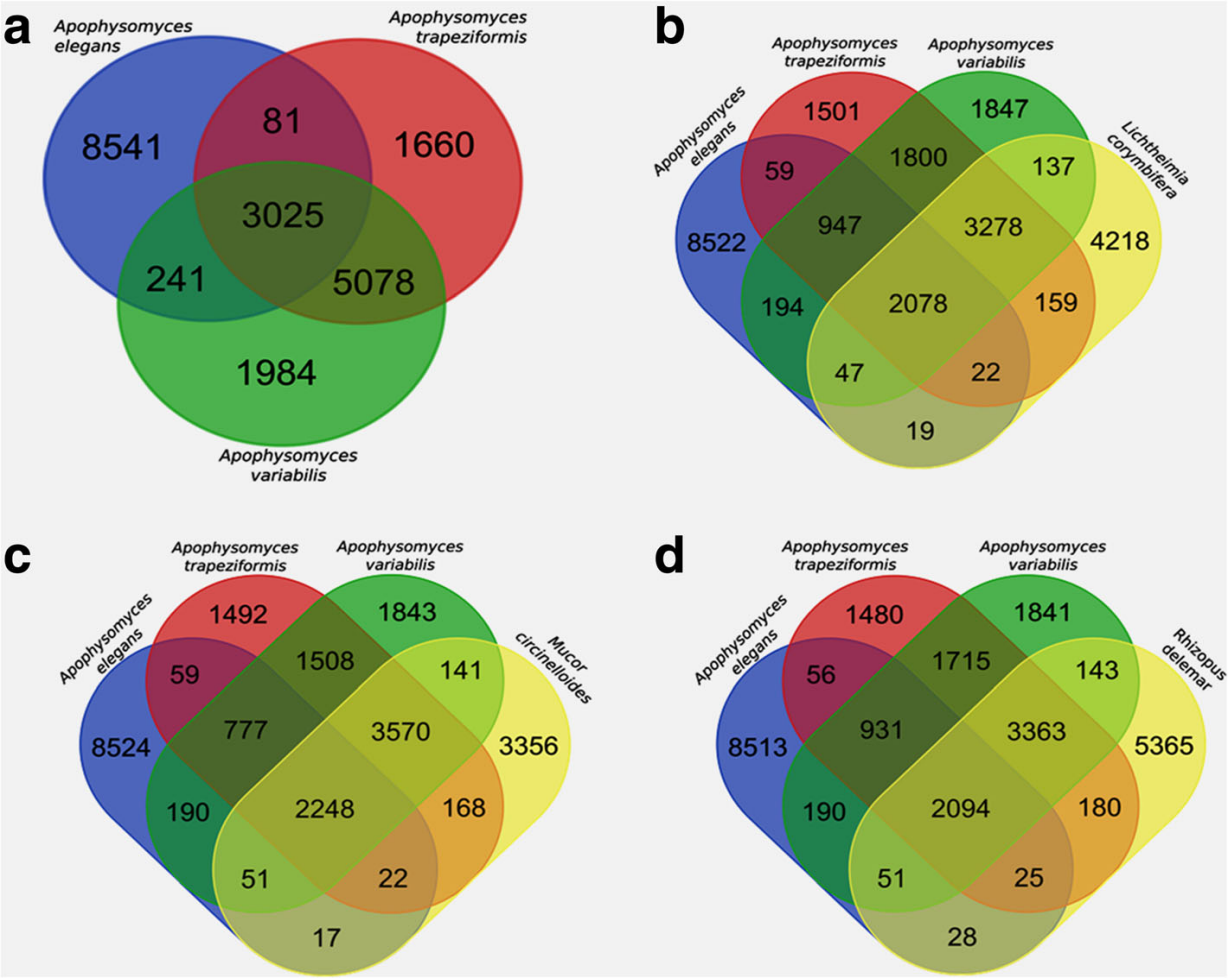
Orthologues gene family comparison of *Apophysomyces* species with other *Mucorales.* Distribution of orthologues genes of *Apophysomyces* species in comparison to *R. delemar*, *L. corymbifera* and *M. circinelloides*. Venn diagram showing the presence of common gene homologs: (**a**) within *Apophysomyces* species, orthologs of *Apophysomyces* genomes are compared against *L. corymbifera* (**b**), *M. circinelloidies* (**c**) and *R. delemar* (**d**)

OrthoMCL analysis was performed for three *Apophysomyces* genomes and compared with other *Mucorales*. A total of 3025 ortholog proteins were shared among three *Apophysomyces* genomes (Fig. 2a). Apart from the shared ortholog genes, 5078 genes were shared between *A. trapeziformis* and *A. variabilis* and 241 genes between *A. elegans* and *A. variabilis* (Fig. 2a). *Apophysomyces* genomes shared common orthologues genes with *L. corymbifera* (2078 genes), *M. circinelloides* (2248 genes) and *R. delemar* (2094 genes) (Fig. 2b, c & d). Paralogues/gene duplication events in *Apophysomyces* were predicted separately for each species using the total protein coding genes. Paralog gene prediction data showed 629 paralog/gene duplication groups for *A. variabilis* comprising 2334 genes, 733 paralog groups for *A. trapeziformis* comprising 2175 genes, and 308 paralog groups with 879 genes for *A. elegans*. Approximately 18% of the genes in *A. variabilis* and *A. trapeziformis* genomes were found to be duplicated and 6.9% in *A. elegans*. The maximum number of gene paralogs had transposons within the genes, which possibly play a role in gene duplications. Several gene expansions had been observed in cell wall components (chitin synthase, chitin deacetylase), electron transport chain (ABC membarane, E1-E2 ATPase, cytochrome p450), secreted proteases (aspartic protease), sugar transporters, CotH invasins and signal transduction pathways in all three *Apophysomyces* species.

### Predicted virulence properties

PHIbase tool was used to determine the possible genes associated with virulence in *Apophysomyces* species. The analysis predicted diverse protein domains, families and repeats encompassing 5.7%, 6.4% and 6.1% genes of *A. variabilis*, *A. trapeziformis* and *A. elegans* respectively for virulence properties of the organisms. The protein kinase domain and Ras proteins were the predominant genes associated with virulence in *Apophysomyces* species. CotH proteins (invasins), proteases, siderophores, CBS domains, cell wall components (chitin synthase, chitin deacetylase) and septins were the other genes determining virulence in *Apophysomyces* species**.**

Like other *Mucorales*, *Apophysomyces* genomes contain multiple copies of CotH protein (42.8 kDa protein). *A. variabilis* and *A. trapeziformis* had 17 copies of CotH genes, whereas *A. elegans* had 16 copies. The orthoMCL analysis revealed multiple groups of CotH paralog genes in three *Apophysomyces* species.

*A. variabilis* genome had 488 predicted protease gene families evaluated from MEROPS database, 468 in *A. trapeziformis* and 422 in *A. elegans* (Table 3). Serine proteases were the major group of proteases found in all *Apophysomyces* genomes ranging from 36.8 to 42.1% of all predicted proteases, followed by metallo-proteases. *A. variabilis* genome revealed two-fold higher expansion of aspartic protease genes (22.1%) compared to 9.2% of *A. elegans* and 13.6% of *A. trapeziformis*. Single cluster of aspartic protease paralog gene was noted in all three species. However, expansion of paralogs genes encoding aspartic protease 2 (PF13650) was observed only in *A. variabilis*. OrthoMCL analysis revealed the presence of transposons within the protein domain of aspartic proteases. This phenomenon suggested the possibility of aspartic protease gene insertion at multiple sites of genome. Subtilase, the major serine proteases in *Apophysomyces* genomes revealed multiple paralog genes; three paralog groups for *A. trapeziformis,* two for *A. variabilis* and one for *A. elegans.* Genes involved in iron acquisition pathways were conserved in *Apophysomyces* genomes (Table 4). *Apophysomyces* species contained high affinity iron permease (FTR1) genes (iron acquisition from host), IucA/IucC and FhuF-like transporter (regulator for siderophore biosynthesis), heme oxygenase (utilisation of heme from blood) and ferritin (iron storage) genes. However, the expansion/duplication of iron acquisition genes (siderophores, heme oxygenase, ferritin, ferrochelatase) was not found in *Apophysomyces* genomes. *Apophysomyces* genomes contained multiple copies of CBS domain (iron binding and acquisition). OrthoMCL analysis did not detect any paralog gene for CBS domain.

**Table 3:** Protease gene families in *Apophysomyces* genomes

Proteases	*A. variabilis* N (%)	*A. elegans* N (%)	*A. trapeziformis* N (%)	*L. corymbifera* ^a^ N (%)
Aspartic protease	109 (22.3)	41 (9.2)	64 (13.6)	60 (14.5)
Cysteine protease	78 (15.9)	85 (19.2)	87 (18.5)	68 (16.5)
Metalloprotease	101 (20.6)	110 (24.8)	111 (23.7)	146 (35.4)
Serine protease	180 (36.8)	186 (42.1)	186 (39.7)	124 (30)
Threonine protease	18 (3.6)	18 (4.1)	18 (3.8)	14 (3.4)
Protease of unknown type	2 (0.04)	2 (0.05)	2 (0.04)	1 (0.2)
Total	488	442	468	413

‘N’ denotes total number of proteases predicted by MEROPS database

^a^The information is collected from Reference No. 22

**Table 4:** Genes responsible for iron uptake mechanism in *Apophysomyces* genomes

Genes	*A. variabilis* (N)	*A. elegans* (N)	*A. trapeziformis* (N)	*L. corymbifera* ^a^(N)
Reductive pathway				
High affinity iron permease (FTR1)	2	2	2	4
Multicopper oxidase	8	10	8	8
Ferric reductase	3	3	3	3
Zinc/iron permease	7	7	8	6
Siderophore transporter				
IucA / IucC family and Ferric iron reductase FhuF-like transporter	2	2	2	1
Heme oxygenase	2	2	2	2
Ferritin	3	3	3	2
Ferrochelatase	1	1	1	–

‘N’ denotes gene copy numbers as annotated by PFAM database

^a^The information is collected from Reference No. 22

### Carbohydrate-active enzymes (CAZymes) in *Apophysomyces* genomes

CAZymes were predicted and classified using dbCAN. *Apophysomyces* genome contained glycoside hydrolases (GHs), glycosyl transferases (GTs), carbohydrate esterases (CEs), carbohydrate binding modules (CBMs) and polysaccharide lyases (PLs) genes (Table 5, Additional file 2: Table S2). *A. variabilis* had 97 glycoside hydrolases (GHs) genes predominantly belonging to GH18 family (chitinases). The glycosyl transferases (GTs) were the major CAZymes found in *A. variabilis*. This class of enzyme largely belongs to GT2 family of chitin synthase genes, an essential component for cell wall synthesis. *A. variabilis* had 75 carbohydrate esterases (CEs) genes and majority consisted of CE4 family that code for chitin deacetylase, the enzyme that helps in the conversion of chitin to chitosan. *A. variabilis* contained other CAZymes like polysaccharide lyases (PLs) and carbohydrate binding modules (CBMs) in their genome. *Apophysomyces* species had similar percentage of CAZymes in their genome (Table 5). The orthoMCL analysis revealed multiple gene duplications/paralogs in CAZymes.

**Table 5:** Comparison between carbohydrate active enzymes (CAZymes) of *Apophysomyces* species and other *Mucorales*

Carbohydrate active enzymes	*A. variabilis* N (%)	*A. elegans* N (%)	*A. trapeziformis* N (%)	*R. arrhizus* ^a^ N (%)	*R. miehei* ^a^ N (%)
Glycoside hydrolase (GHs)	97 (28.2)	92 (27.1)	95 (26.9)	118 (35.4)	110 (40.7)
Glycosyl transferase (GTs)	118 (34.3)	113 (33.2)	125 (35.4)	143 (42.9)	118 (43.7)
Carbohydrate esterase (CEs)	75 (21.8)	81 (23.8)	76 (21.5)	39 (11.7)	20 (7.4)
Carbohydrate-binding module (CBMs)	51 (14.8)	51 (15)	54 (15.3)	27 (8.1)	20 (7.4)
Polysaccharide lyase (PLs)	3 (0.87)	3 (0.88)	3 (0.85)	6 (1.8)	2 (0.74)
Total	344	340	353	333	270

‘N’ denotes number of CAZymes as predicted by dbCAN database

^a^ The information is collected from Reference No. 23

### Secretory protein analysis

A total of 435 (3.3%) genes encoding the secretory proteins were predicted for *A. elegans*, 435 (3.6%) genes for *A. trapeziformis* and 412 (3.2%) genes for *A. variabilis*. The predicted secretory proteins of *Apophysomyces* genome predominantly contained carbohydrate active enzymes. The diverse group of secreted CAZymes in *Apophysomyces* genome were glycoside hydrolase (GHs) that consisted of chitinases (GH 18) and lysozymes (GH 25), followed by carbohydrate esterase (CEs) that predominantly consisted of chitin deacetylase (CE4). To predict the secretory peptidases, the secretory proteins were searched against MEROPS peptidase database using BLASTp. Aspartic protease and subtilase were the major peptidases in *Apophysomyces* genome and those enzymes might play role in tissue destruction of the host. BLASTp analysis against lipase engineering data base revealed 9 to 14 genes coding for secretory lipase in the *Apophysomyces* species (Table 6).

**Table 6:** Predicted secretory proteins from *Apophysomyces* genomes

Secretory Proteins	*A. elegans*	*A. trapeziformis*	*A. variabilis*
Number of CAZymes	68	66	66
Carbohydrate-binding module (CBMs)	11	14	10
Carbohydrate esterase (CEs)	20	19	20
Glycoside hydrolase (GHs)	28	23	30
Glycosyl transferase (GTs)	8	9	5
Polysaccharide lyase (PLs)	1	1	1
Number of Proteases	36	32	41
Aspartic protease	12	12	14
Cysteine protease	2	2	2
Metalloprotease	4	3	5
Serine protease	18	15	20
Number of Lipases	14	9	10

### Phylogenetic analysis

**Fig. 3:**
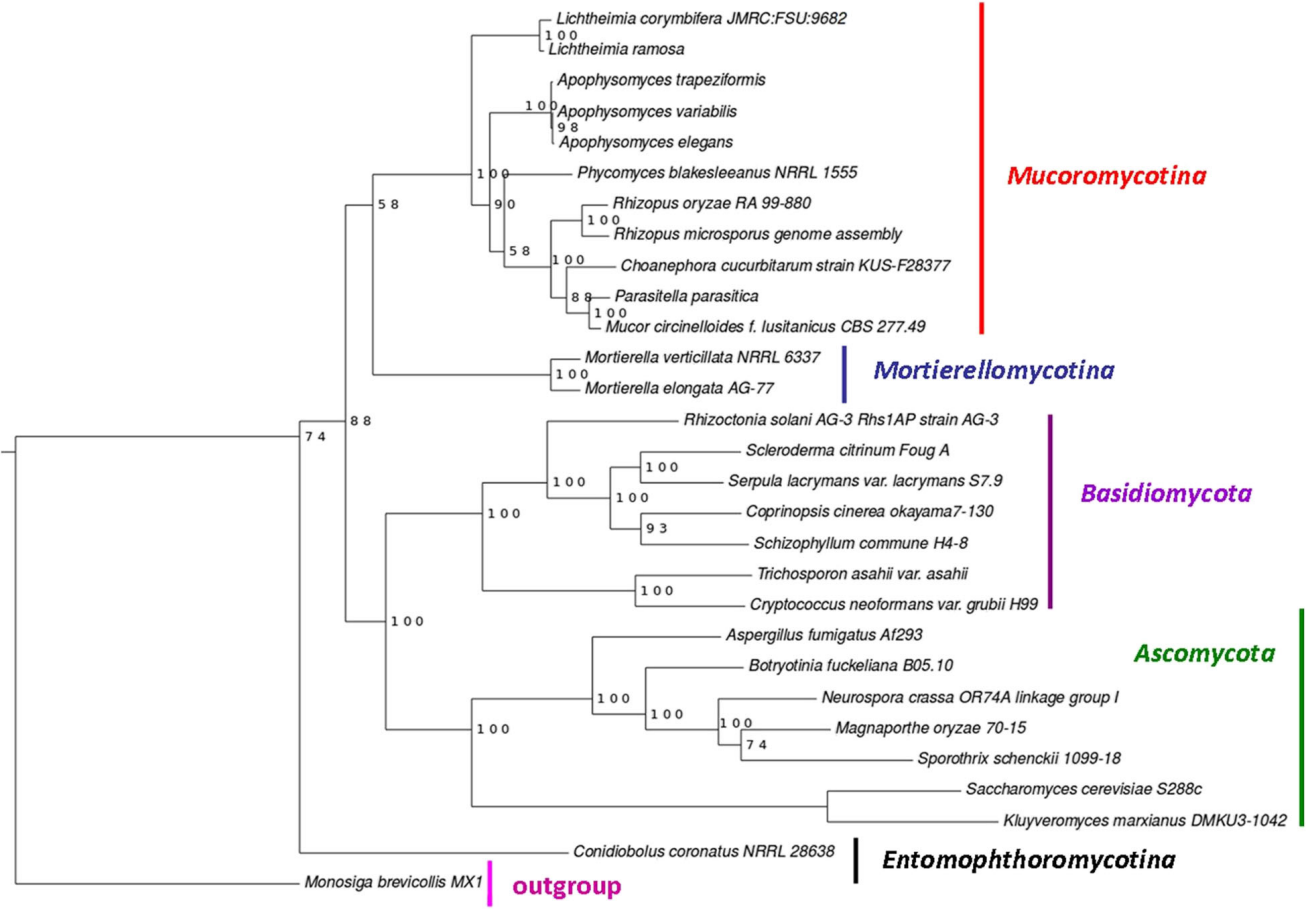
Molecular phylogenetic analysis of *Apophysomyces* species. Phylogenetic tree constructed using 13 single copy genes from 29 fungal species by LG amino acid substitution model with 100 bootstrap replications

We used BUSCO to identify near universal single-copy ortholog proteins from 29 species. A total of 13 single copy gene proteins, common to all 29 species were identified and used for phylogenetic tree construction (Table 7). The fungal species were classified into two major groups, *Ascomycota* and *Basidiomycota*. Fungi belonging to the subkingdom *Mucoromycotina* were closely related to *Mortierellomycotina*. The *Mucorales* were grouped in two major clusters. The three *Apophysomyces* species were grouped together in close association with *Phycomyces blakesleeanus* (Fig. 3). A separate phylogenetic tree was constructed by excluding *Ascomycota* and *Basidiomycota*, to increase the single copy genes. A total of 72 single copy genes from *Mucoromycotina*, *Mortierellomycotina* and *Entomophthoromycotina* were analysed, which revealed similar findings as that of 13 single copy genes (Additional file 1: Figure S2).

**Table 7:** List of 13 single copy genes/protein sequences used in the phylogenetic analysis of *Apophysomyces* species

S. No	BUSCO IDs	Protein gene family
1	BUSCOfEOG7CVQ76	Orotidine 5′ phosphate decarboxylase
2	BUSCOfEOG7DFXNZ	Nucleolar GTP-binding protein 2
3	BUSCOfEOG7DZ8XZ	Transcription initiation factor TFIID subunit 9
4	BUSCOfEOG7FJH8G	Phosphoribosylaminoimidazole carboxylase
5	BUSCOfEOG7TJ3V6	Proteosome core subunit beta 2 (PUP1)
6	BUSCOfEOG7WDNCV	Serine/threonine protein kinase (RIO1)
7	BUSCOfEOG7ZWDC9	1-(5-phosphoribosyl)-5-[(5-phosphoribosylamino)methylideneamino] imidazole-4-carboxamide isomerase
8	BUSCOfEOG70KGZ3	Proteasome regulatory particle base subunit (RPN1)
9	BUSCOfEOG71VT2Q	Pre-mRNA-splicing factor (CWC22)
10	BUSCOfEOG74R219	Signal recognition particle receptor subunit alpha (SRP101)
11	BUSCOfEOG78SQSW	Glycylpeptide N-tetradecanoyltransferase
12	BUSCOfEOG78WM1Q	Multifunctional tryptophan biosynthesis protein (anthranilate synthase component 2)
13	BUSCOfEOG779P7X	Octanoyltransferase

## Discussion

The current study revealed *A. variabilis* genome of 39.38 Mb. The genome was compared with available genome sequences of *A. elegans* (38.46 Mb) and *A. trapeziformis* (35.84 Mb). The genome structures of three pathogenic *Apophysomyces* species were compared with other pathogenic *Mucorales* to identify the predicted virulence properties of *Apophysomyces* species. *Apophysomyces* species have more than 12,000 protein coding genes. Among *Mucorales*, *Mucor racemosus* has the maximum number of protein coding genes (21,385 genes) with genome size of 75.32 Mbs and *Umbelopsis isabellina* has the least number of protein coding genes (7612 genes) with genome size of 21.87 Mbs [20]. The protein coding genes of common human pathogens, *Rhizopus arrhizus* (17,467 genes) and *Lichtheimia corymbifera* (12,379 genes) lie close to *Apophysomyces* species [21, 22]. The predicted protein families of *Apophysomyces* species (~1800 protein families), is also close to *Rhizomucor miehei* (1827 proteins) and *R. arrhizus* (1653 proteins) [23]. Phylogenetically, *Mucoromycotina* including *Apophysomyces* species is closely related to *Mortierellomycotina* rather than *Entomophthoromycotina.* The phylogenetic analysis of the present study confirms the findings of super tree constructed of 9748 genes using parsimony approach [22]. The present findings also confirm that pathogenic *Mucorales* are genetically different from *Entomophthorales* [20].

The transposable elements (TEs) in the fungal genomes play a major role in the genomic diversity and genome evolution [24]. The presence of TEs in fungi under *Ascomycetes* and *Basidiomycetes* leads to high degree of diversity ranging from 0.02 to 29.8% of their genomes [24]. *Mucorales* also have a varying percentage of transposable elements in their genomes ranging from 3.17% (*Rhizomucor miehei*) to 35% (*P. blakesleeanus* and *Absidia glauca*) [21, 23]. The present analysis showed that *Apophysomyces* species have lower content of TEs in their genome compared to other *Mucorales.* The lower content of TEs in *L. corymbifera* was associated with the appearance of hetereokaryon incompatibility (HET) genes and RNA interference mediated RNA degradation genes like dicer, argonaute and translation initiation factor 2C [22]. Similarly, *Apophysomyces* species contain multiple copies of HET genes in their genome along with RNA interference mediated genes. In comparison, *R. arrhizus* has 35% of TEs in their genome, which may be due to the absence of HET genes [21]. The HET domain is found in all fungi under *Ascomycetes* and helps in the formation of heterokaryons in sexual reproduction. The function of HET domain in *Lichtheimia* and *Apophysomyces* species is not clearly known. The genome duplication and transposon mediated gene expansion may facilitate the pathogenic mechanism of fungi [25].

In Kingdom Fungi, the whole genome duplication has been identified in two separate group of fungi: *Saccharomycotina*, an ancestry of the Dikarya [26, 27] and in *R. arrhizus* [21]. In *R. arrhizus*, the whole genome duplication is an important phenomenon for the expansion of multiple gene families and plays significant role in pathogenesis and antifungal resistance of the organism [21, 28]. The presence of two copies of *RNase P* may attribute for whole genome duplication in *R. arrhizus* [21]. The analysis of *L. corymbifera* genome showed the presence of tandem and segmental gene duplication [22]. Single copy of *RNase P* was found in *Apophysomyces* species like *L. corymbifera* and indicate the possibility of tandem and segmental duplications in *Apophysomyces* genomes. *Mucorales* in general contain more duplicated genome regions (average of 4 to 13 genes) compared to other fungal genomes [29]. The duplication of genes coding for signal transduction pathways (protein kinase, ATP binding) and transport components have been reported in mucoralean genomes [29]. Multiple gene expansion were noticed in *Apophysomyces* genomes for ABC membrane transport, major facilitator proteins, E1-E2 ATPase and other protein families that were involved in cell wall synthesis, morphogenesis, virulence mechanism and antifungal resistance.

The pathogen-host interaction database predicted approximately 6% of genes in *Apophysomyces* genomes are associated with virulence of the fungi. The multiple copies of protein kinase and Ras domain in *Apophysomyces* genomes may play a role in pathogenesis. Protein kinase plays a major role in signal transduction pathways and regulates major cellular functions, cell cycle regulation and fungal morphogenesis [30]. Two-fold rise in the protein kinase gene family has been observed in *R. arrhizus*. Similarly, the present analysis showed *Apophysomyces* genomes had multiple paralog gene clusters of protein kinase. The Ras protein regulates eukaryotic growth and differentiation, and orchestrates virulence in pathogenic fungi [31].

The most common virulence trait of *Mucorales* is iron acquisition from the host. The mechanisms included reductive pathways consisting of high affinity iron permease (FTR1), multi-copper oxidase and ferric reductase, siderophore transporter and heme oxygenase [32–34]. In normal hosts, iron in blood is bound to carrier proteins like transferrin, ferrin and lactoferrin. The bound form of iron limits the toxic effects and maintains iron homeostasis [33]. During diabetic ketoacidosis (DKA), the binding affinity of iron to the carrier protein is reduced, leading to the release of free iron in the blood. Free iron helps *Mucorales* to grow in the body [35]. In *L. corymbifera*, up-regulation of FTR1 gene, multi-copper oxidase and ferric reductase is observed under iron starvation [22]. The reduction in the copy number of FTR1 gene abolishes the iron uptake from ferroxamine in *R. arrhizus* [35]. These findings suggest the role of reductase/permease pathway in iron uptake among *Mucorales*. *Apophysomyces* genome contains all the essential components of reductive pathways of high affinity iron permease (FTR1), multi-copper oxidase and ferric reductase that help the iron acquisition from the host. Like other *Mucorales*, *A. variabilis* lacks hydroxamate siderophores (NRPS genes) in their genome. However, compensatory mechanism has been observed in *L. corymbifera,* in the form of upregulation of IucA/IucC family and ferric iron reductase FhuF-like transporter (bacterial siderophores) during iron starvation [22]. *Apophysomyces* genomes also contain genes of IucA/IucC family and ferric iron reductase FhuF-like transporter. The other modes of iron acquisition genes including heme oxygenase pathway and ferritin genes are conserved in *Apophysomyces* species.

Deferoxamine therapy is a risk factor for mucormycosis. Deferoxamine acts as a xenosiderophore and helps in the iron acquisition of *R. arrhizus* [33, 34]. *R. arrhizus* contains CBS domain (Fob1/Fob2 proteins) on their cell surface, which acts as receptor to bind ferroxamine. In deferoxamine treated mice, mutants of Fob proteins have reduced iron uptake, germination and virulence [36]. *Apophysomyces* genome contains multiple copies of CBS domain, which may also play a role in the iron acquisition.

The angio-invasion and tissue necrosis are notable features in mucormycosis infections. The endothelial cell receptors play major role in the angio-invasion of *Mucorales* especially in patients with elevated serum iron concentrations (patients with diabetic ketoacidosis or on deferoxamine therapy) [33, 34]. The glucose-regulated protein-78 (GRP78) receptor on the human endothelial cell is up-regulated in the presence of iron or glucose leading to increase adherence and endocytosis of *R. arrhizus* spores [37]. CotH (invasin) protein on the surface of spore, helps in the binding of the spores to the endothelial cells [20]. The binding of CotH proteins to the GRP78 receptors mediates invasion and endothelial damage [37, 38]. Blocking of CotH receptor with anti CotH antibody abolishes their ability to adhere and invade the host cells [38]. *Apophysomyces* genome possesses multiple copies of CotH genes, which may help in angio-invasion of the fungus. Gene duplication of CotH gene families may increase the virulence properties of *Apophysomyces* species. *Entomopthorales* does not contain CotH genes and has low angio-invasive property [20].

Proteases are also considered to be important for virulence in fungi, as they are responsible for tissue destruction and necrosis in hosts. The genome of *A. variabilis* contains 488 predicted proteases (3.8%), closely similar to *R. arrhizus* (3.6%) and *L. corymbifera* (3.3%) [22]. The common secretory proteases of *A. variabilis* are serine (48.7%) and aspartic protease (34%). The number of secretory protease in *Apophysomyces* genome (8%) is similar to that of *R. delemar* (7%) [22]. However, unlike *R. arrhizus*, *Apophysomyces* species can cause severe tissue destruction in the immunocompetent host.

*Mucorales* cell wall contains unique polysaccharides molecules such as chitin and chitosan, which are not seen in mammals [39]. The *Apophysomyces* genome contains β-1,3-glucan synthase genes (FKS1), but α-1,3-glucan synthase genes are absent. Despite the presence of FKS1 gene, the inherent resistance mechanism of *Mucorales* to echinocandins is not clear [29]. CAZymes play important role in biological processes including cell wall synthesis, cell signaling and energy production [40]. The CAZymes constitute the majority of the secretory proteins in the *Apophysomyces* genomes (Table 6). However, *Apophysomyces* contains lower amount of glycosyl hydrolases and glycosyl transferase compared to *R. arrhizus* and *R. miehei* (Table 5). The expansion in the gene clusters coding for carbohydrate esterase (CEs) and carbohydrate-binding module (CBMs) possibly compensates those deficiencies in *Apophysomyces* species (Table 5)**.** The most common CEs in *Apophysomyces* genomes are CE10 (carboxyl esterase) and CE4 (chitin deacetylase), but CE10 is absent in *R. arrhizus* and *R. miehei* [23]. The expansion of cell wall genes, chitin deacetylase and chitin synthase genes are observed in all the *Mucorales*. However, the functional consequences of these genes are not clear [20–22, 29].

## Conclusion

We report the maiden draft genome sequence of *A. variabilis,* a common pathogen causing mucormycosis in Indian subcontinent. The genome analysis of *A. variabilis* together with other two pathogenic *Apophysomyces* species revealed multiple gene duplications in signal transduction pathways, cell signalling molecules and virulence traits. The expansion of virulence genes such as secretory protease, CotH invasins, cell wall components (chitin synthase and chitin deacetylase) is seen in *Apophysomyces* genomes. The unique CAZymes responsible for the biosynthesis of cell wall polysaccharides may be future antifungal target. Further exploration of *Mucorales* specific cell wall components may help in understanding the pathogenesis of mucormycosis in detail.

## Methods

### Fungal strain

*Apophysomyces variabilis* strain NCCPF 102052 isolated from patient with necrotising fasciitis of thigh was retrieved from the National culture collection for pathogenic fungi (NCCPF), Chandigarh, India. The isolate was grown on potato dextrose agar by incubating at 35 °C for 7 days. DNA was isolated from the fresh mycelia using phenol: chloroform: isoamyl alcohol extraction protocol as described earlier [41].

### Genome sequencing, assembly and annotation

The whole genome sequence of *A. variabilis* NCCPF 102052 was performed using paired-end (PE)/(MP) 2*150/2*125 base pair library on Hiseq platform (Illumina, San Diego, California, USA). The paired-end sequencing library was prepared using Illumina TruSeq Nano DNA HT library preparation kit and the mean size of the library was 645 bp. The Illumina mate- pair libraries were prepared using Cre-Lox recombination method with insert size of 3-5Kb [42]. The raw data was filtered using trimmomatic v0.30 [43] and per base sequence quality score (Q) ≥20 was considered. High quality data were assembled using Soapdenovo2 assembler as described by Li et al., to generate scaffolds using optimized parameters [44, 45]. The whole genome sequence of *Apophysomyces trapeziformis* (B9324, GenBank accession ID: JNDP00000000.1) and *Apophysomyces elegans* (B7760, GenBank accession ID: JNDQ00000000.1) were downloaded from NCBI genome database for the comparative analysis.

### Gene prediction

AUGUSTUS version–3.1.01 was used for ab initio gene prediction [46]. *Rhizopus arrhizus* training set was used as species for gene prediction from both strands. Prediction of incomplete genes was allowed. Using this gene model, CDS & protein sequence was extracted. Protein coding genes were annotated using BLASTx program available from NCBI [47]. Best BLAST hit annotation was transferred to the query protein when the E-value was less than or equal to 10^-5 and identity greater than or equal to 40%. Swiss-Prot database was used for homology searches [48].Based on these annotations, Gene ontology (GO) and KEGG annotation were retrieved for individual proteins wherever applicable [49].

### Protein family annotation

PFAM annotation was performed using PFAM scan tool downloaded from PFAM database [50]. This tool was used for scanning against PFAM database version 30.03 [51].

### Transposable elements

TransposonPSI was used to identify diverse families of transposable elements that prevail in the genome sequences. The transposons were classified into DNA and RNA transposons [47].

### Non-coding RNA

Non-coding-RNAs were identified and annotated using infernal tool of RFAM database [52].

### Peptidase prediction

Peptidase analysis was conducted using BLASTp search against MEROPS database [53]. Annotations were transferred to the query proteins if the identity between query and target proteins were at-least 40% and *e* value = 10^-4.

### Carbohydrate active enzyme (CAZymes) prediction

CAZymes annotation was done using dbCAN database [40]. Hmmscan version 3.1 was used to search against dbCAN and in-house scripts were used to identify the best hit.

### Secretory protein analysis

The soluble secretory proteins satisfying the following parameters were analysed: a protein with N-terminal signal peptide, without the presence of trans-membrane domain, complete absence of GPI-anchor site and extracellular secretion of the proteins outside the cell [54, 55]. Secreted proteins were predicted using a customised bioinformatic pipeline including signalP (version 4.1) and TMHMM (version 2.0) software [56, 57]*.* The presence of signal peptides in the protein sequence was predicted by setting the D-cut off values to “sensitive” (option eukaryotic). The parameter setup includes that secreted proteins that did not contain any transmembrane helix or the one overlapping the signal peptide within first 40 amino acids of the protein sequences. TargetP software (version 1.1) and WolfPsort (version 0.2) were used to predict protein subcellular localization. Proteins were considered secretory if subcellular localization was assigned to extracellular secretory pathway [58, 59]. PS-SCAN (PS00014) was used to find endoplasmic reticulum localisation motif [60]. The predicted secreted proteins were annotated using BLASTp query search (*e* value = 10^-5): dbCAN was used to predict CAZymes, MEROPS for peptidase and Lipase Engineering Database for lipase prediction [40, 53, 61].

### Pathogen-host interaction (PHI) gene prediction

Genes responsible for possible pathogenesis and virulence mechanism were identified and annotated using PHI-base [62]. BLASTp was used to search for similar sequences from PHI-base and the annotation from best hit annotated sequence was transferred to the query proteins.

### OrthoMCL analysis for ortholog and paralog gene prediction

Based on sequence similarity and graph algorithm, OrthoMCL was used to determine the orthologs, co-orthologs and paralogs. Orthologs and co-orthologs are the speciation events and paralogs describe the duplication event within a genome [63, 64]. For OrthoMCL analysis a total of six genomes including three *Apophysomyces* genomes, one genome each of *R. arrhizus, L. corymbifera* and *Rhizomucor miehei* were used (Additional file 2: Table S3).

### Phylogenetic analysis of *Apophysomyces* species using whole genome sequences

For phylogenetic analysis, 29 species under different genera from *Ascomycota*, *Basidiomycota*, *Mucoromycotina*, *Mortierellomycotina* and *Entomophthoromycotina* were included in the analysis (Additional file 2: Table S3). For phylogenetic tree construction, a single copy orthologous protein was identified using BUSCO [65]. The common proteins were used for multiple sequence alignment by MAFFT [66]. Only conserved regions were considered for further analysis by removing the non-conserved regions using Gblocks [67]. Conserved regions of different single copy proteins from each species were concatenated and then subjected to phylogenetic tree construction using PhyML and LG model [68, 69].

## Additional files

Additional file 1: Figure S1. Gene ontology (GO) of *Apophysomyces* genomes. **Figure S2**. Phylogenetic analysis of *Apophysomyces* species with other *Mucorales*. (DOC 376 kb)

Additional file 2: Table S1. Non coding RNAs in genomes of three *Apophysomyces* species. **Table S2**. Carbohydrate active enzymes (CAZymes) of *Apophysomyces* species. **Table S3**. GenBank accession numbers of whole genome sequences used in phylogenetic analysis of *Apophysomyces* species and orthoMCL analysis. (DOC 163 kb)

## Abbreviations

CAZymes: carbohydrate active enzymes
GO: Gene ontology
KEGG: Kyoto encyclopedia of genes and genomes
LG model: Le & Gascuel model
TE: Transposable elements

## References

[1] Hibbett DS, Binder M, Bischoff JF, Blackwell M, Cannon PF, Eriksson OE (2007). A higher-level phylogenetic classification of the fungi. Mycol Res.

[2] Walther G, Pawłowska J, Alastruey-Izquierdo A, Wrzosek M, Rodriguez-Tudela JL, Dolatabadi S (2013). DNA barcoding in Mucorales: an inventory of biodiversity. Persoonia.

[3] Hoffmann K, Pawłowska J, Walther G, Wrzosek M, de Hoog GS, Benny GL (2013). The family structure of the Mucorales: a synoptic revision based on comprehensive multigene-genealogies. Persoonia.

[4] Spatafora JW, Chang Y, Benny GL, Lazarus K, Smith ME, Berbee ML (2016). A phylum-level phylogenetic classification of zygomycete fungi based on genome-scale data. Mycologia.

[5] Ribes JA, Vanover-Sams CL, Baker DJ (2000). Zygomycetes in human disease. Clin Microbiol Rev.

[6] Roden MM, Zaoutis TE, Buchanan WL, Knudsen TA, Sarkisova TA, Schaufele RL (2005). Epidemiology and outcome of zygomycosis: a review of 929 reported cases. Clin Infect Dis.

[7] Skiada A, Pagano L, Groll A, Zimmerli S, Dupont B, Lagrou K (2011). Zygomycosis in Europe: analysis of 230 cases accrued by the registry of the European Confederation of Medical Mycology (ECMM) working group on Zygomycosis between 2005 and 2007. Clin Microbiol Infect.

[8] Petrikkos G, Skiada A, Drogari-Apiranthitou M (2014). Epidemiology of mucormycosis in Europe. Clin Microbiol Infect.

[9] Chakrabarti A, Chatterjee SS, Das A, Panda N, Shivaprakash MR, Kaur A (2009). Invasive zygomycosis in India: experience in a tertiary care hospital. Postgrad Med J.

[10] Bala K, Chander J, Handa U, Punia RS, Attri AK (2015). A prospective study of mucormycosis in north India: experience from a tertiary care hospital. Med Mycol.

[11] Chakrabarti A, Shivaprakash MR, Curfs-Breuker I, Baghela A, Klaassen CH, Meis JF (2010). Apophysomyces elegans: epidemiology, amplified fragment length polymorphism typing, and in vitro antifungal susceptibility pattern. J Clin Microbiol.

[12] Misra PC, Srivastava KJ, Lata K (1979). Apophysomyces, a new genus of the Mucorales. Mycotaxon.

[13] Prakash H, Ghosh A, Rudramurthy S, Paul R, Gupta S, Negi V (2016). The environmental source of emerging apophysomyces variabilis infection in india. Med Mycol.

[14] Alvarez E, Stchigel AM, Cano J, Sutton DA, Fothergill AW, Chander J (2010). Molecular phylogenetic diversity of the emerging mucoralean fungus Apophysomyces: proposal of three new species. Rev Iberoam Micol.

[15] Bonifaz A, Stchigel AM, Guarro J, Guevara E, Pintos L, Sanchis M (2014). Primary cutaneous mucormycosis produced by the new species Apophysomyces mexicanus. J Clin Microbiol.

[16] Guarro J, Chander J, Alvarez E, Stchigel AM, Robin K, Dalal U (2011). Apophysomyces variabilis infections in humans. Emerg Infect Dis.

[17] Chander J, Miguel A, Alastruey-izquierdo A, Jayant M, Bala K, Rani H (2015). Fungal necrotizing fasciitis, an emerging infectious disease caused by Apophysomyces ( Mucorales ). Rev Iberoam Micol.

[18] Etienne KA, Gillece J, Hilsabeck R, Schupp JM, Colman R, Lockhart SR (2012). Whole genome sequence typing to investigate the Apophysomyces outbreak following a tornado in Joplin, Missouri, 2011. PLoS One.

[19] Neblett Fanfair R, Benedict K, Bos J, Bennett SD, Lo Y-C, Adebanjo T (2012). Necrotizing cutaneous mucormycosis after a tornado in Joplin, Missouri, in 2011. N Engl J Med.

[20] Chibucos MC, Soliman S, Gebremariam T, Lee H, Daugherty S, Orvis J (2016). An integrated genomic and transcriptomic survey of mucormycosis-causing fungi. Nat Commun.

[21] Ma LJ, Ibrahim AS, Skory C, Grabherr MG, Burger G, Butler M (2009). Genomic analysis of the basal lineage fungus Rhizopus oryzae reveals a whole-genome duplication. PLoS Genet.

[22] Schwartze VU, Winter S, Shelest E, Marcet-Houben M, Horn F, Wehner S (2014). Gene expansion shapes genome architecture in the human pathogen Lichtheimia corymbifera: an evolutionary genomics analysis in the ancient terrestrial mucorales (Mucoromycotina). PLoS Genet.

[23] Zhou P, Zhang G, Chen S, Jiang Z, Tang Y, Henrissat B (2014). Genome sequence and transcriptome analyses of the thermophilic zygomycete fungus Rhizomucor miehei. BMC Genomics.

[24] Castanera R, López-Varas L, Borgognone A, LaButti K, Lapidus A, Schmutz J (2016). Transposable elements versus the fungal genome: impact on whole-genome architecture and transcriptional profiles. PLoS Genet.

[25] Santana MF, Queiroz MV (2015). Transposable elements in fungi: a genomic approach. Scientific J Genetics Gen Ther.

[26] Kellis M, Birren BW, Lander ES (2004). Proof and evolutionary analysis of ancient genome duplication in the yeast Saccharomyces Cerevisiae. Nature.

[27] Wolfe KH, Shields DC (1997). Molecular evidence for an ancient duplication of the entire yeast genome. Nature.

[28] Lewis RE, Lortholary O, Spellberg B, Roilides E, Kontoyiannis DP, Walsh TJ (2012). How does antifungal pharmacology differ for mucormycosis versus aspergillosis?. Clin Infect Dis.

[29] Corrochano LM, Kuo A, Marcet-Houben M, Polaino S, Salamov A, Villalobos-Escobedo JM (2016). Expansion of signal transduction pathways in fungi by extensive genome duplication. Curr Biol.

[30] Kosti I, Mandel-Gutfreund Y, Glaser F, Horwitz BA (2010). Comparative analysis of fungal protein kinases and associated domains. BMC Genomics.

[31] Fortwendel JR (2015). Orchestration of morphogenesis in filamentous fungi: conserved roles for Ras signaling networks. Fungal Biol Rev.

[32] Spellberg B, Edwards J, Ibrahim A (2005). Novel perspectives on mucormycosis: pathophysiology, presentation, and management. Clin Microbiol Rev.

[33] Ibrahim AS (2011). Host cell invasion in mucormycosis: role of iron. Curr Opin Microbiol.

[34] Ibrahim A, Spellberg B, Edwards JJ (2008). Iron acquisition: a novel prospective on Mucormycosis pathogenesis and treatment. Curr Opin Infect Dis.

[35] Ibrahim AS, Gebremariam T, Lin L, Luo G, Husseiny MI, Skory CD (2010). The high affinity iron permease is a key virulence factor required for Rhizopus oryzae pathogenesis. Mol Microbiol.

[36] Liu M, Lin L, Gebremariam T, Luo G, Skory CD, French SW (2015). Fob1 And Fob2 proteins are virulence determinants of Rhizopus oryzae via facilitating iron uptake from Ferrioxamine. PLoS Pathog.

[37] Liu M, Spellberg B, Phan QT, Fu YY, Fu YY, Lee AS (2010). The endothelial cell receptor GRP78 is required for mucormycosis pathogenesis in diabetic mice. J Clin Invest.

[38] Gebremariam T, Liu M, Luo G, Bruno V, Phan QT, Waring AJ (2014). CotH3 Mediates fungal invasion of host cells during mucormycosis. J Clin Invest.

[39] Mélida H, Sain D, Stajich JE, Bulone V (2015). Deciphering the uniqueness of Mucoromycotina cell walls by combining biochemical and phylogenomic approaches. Environ Microbiol.

[40] Yin Y, Mao X, Yang J, Chen X, Mao F, Xu Y (2012). DbCAN: a web resource for automated carbohydrate-active enzyme annotation. Nucleic Acids Res.

[41] Lee SB, Taylor JW, Innis MA, Gelfand DH, Sninsky JJ, White TJ (1990). Isolation of DNA from fungal mycelia and single spores. PCR protocols: a guide to methods and applications.

[42] Van Nieuwerburgh F, Thompson RC, Ledesma J, Deforce D, Gaasterland T, Ordoukhanian P (2012). Illumina mate-paired DNA sequencing-library preparation using Cre-lox recombination. Nucleic Acids Res.

[43] Bolger AM, Lohse M, Usadel B (2014). Trimmomatic: a flexible trimmer for Illumina sequence data. Bioinformatics.

[44] Luo R, Liu B, Xie Y, Li Z, Huang W, Yuan J (2012). SOAPdenovo2: An empirically improved memory-efficient short-read de novo assembler. Gigascience.

[45] Li R, Zhu H, Ruan J, Qian W, Fang X, Shi Z, et al. De novo assembly of human genomes with massively parallel short read sequencing. Genome Res. 2010:265–72. 10.1101/gr.097261.109 PMC2813482 20019144

[46] Stanke M, Steinkamp R, Waack S, Morgenstern B (2004). AUGUSTUS: a web server for gene finding in eukaryotes. Nucleic Acids Res.

[47] Altschul SF, Madden TL, Schäffer AA, Zhang J, Zhang Z, Miller W (1997). Gapped BLAST and PSI-BLAST:a new generation of protein database search programs. Nucleic Acids Res.

[48] Boutet E, Lieberherr D, Tognolli M, Schneider M, Bansal P, Bridge AJ (2016). UniProtKB/Swiss-Prot, the manually annotated section of the UniProt KnowledgeBase: how to use the entry view. Methods Mol Biol.

[49] Ashburner M, Ball CA, Blake JA, Botstein D, Butler H, Cherry JM (2000). Gene ontology: tool for the unification of biology. The Gene Ontology Consortium Nat Genet.

[50] Mistry J, Bateman A, Finn RD (2007). Predicting active site residue annotations in the Pfam database. BMC Bioinformatics.

[51] Finn RD, Coggill P, Eberhardt RY, Eddy SR, Mistry J, Mitchell AL (2016). The Pfam protein families database: towards a more sustainable future. Nucleic Acids Res.

[52] Nawrocki EP, Burge SW, Bateman A, Daub J, Eberhardt RY, Eddy SR (2015). Rfam 12.0: Updates to the RNA families database. Nucleic Acids Res.

[53] Rawlings ND, Barrett AJ, Finn R (2016). Twenty years of the MEROPS database of proteolytic enzymes, their substrates and inhibitors. Nucleic Acids Res.

[54] Lee SA, Wormsley S, Kamoun S, Lee AFS, Joiner K, Wong B (2003). An analysis of the Candida Albicans genome database for soluble secreted proteins using computer-based prediction algorithms. Yeast.

[55] Pellegrin C, Morin E, Martin FM, Veneault-Fourrey C (2015). Comparative analysis of secretomes from ectomycorrhizal fungi with an emphasis on small-secreted proteins. Front Microbiol.

[56] Petersen TN, Brunak S, von Heijne G, Nielsen H (2011). SignalP 4.0: Discriminating signal peptides from transmembrane regions. Nat Methods.

[57] Krogh A, Larsson B, von Heijne G, Sonnhammer EL (2001). Predicting transmembrane protein topology with a hidden Markov model: application to complete genomes. J Mol Biol.

[58] Emanuelsson O, Brunak S, von Heijne G, Nielsen H (2007). Locating proteins in the cell using TargetP, SignalP and related tools. Nat Protoc.

[59] de Castro E, Sigrist CJA, Gattiker A, Bulliard V, Langendijk-Genevaux PS, Gasteiger E (2006). ScanProsite: detection of PROSITE signature matches and ProRule-associated functional and structural residues in proteins. Nucleic Acids Res.

[60] Sigrist CJA, de Castro E, Cerutti L, Cuche BA, Hulo N, Bridge A (2013). New and continuing developments at PROSITE. Nucleic Acids Res.

[61] Fischer M, Pleiss J (2003). The lipase engineering database: a navigation and analysis tool for protein families. Nucleic Acids Res.

[62] Winnenburg R, Baldwin TK, Urban M, Rawlings C, Köhler J, Hammond-Kosack KE (2006). PHI-base: a new database for pathogen host interactions. Nucleic Acids Res.

[63] Chen F, Mackey AJ, Stoeckert CJ, Roos DS (2006). OrthoMCL-DB: querying a comprehensive multi-species collection of ortholog groups. Nucleic Acids Res.

[64] Li L, Stoeckert CJJ, Roos DS (2003). OrthoMCL: identification of Ortholog groups for eukaryotic genomes. Genome Res.

[65] Simão FA, Waterhouse RM, Ioannidis P, Kriventseva EV, Zdobnov EM (2015). BUSCO: assessing genome assembly and annotation completeness with single-copy orthologs. Bioinformatics.

[66] Katoh K, Misawa K, Kuma K, Miyata T (2002). MAFFT: a novel method for rapid multiple sequence alignment based on fast Fourier transform. Nucleic Acids Res.

[67] Castresana J (2000). Selection of conserved blocks from multiple alignments for their use in Phylogenetic analysis. Mol Biol Evol.

[68] Guindon S, Dufayard JF, Lefort V, Anisimova M, Hordijk W, Gascuel O (2010). New algorithms and methods to estimate maximum-likelihood phylogenies: assessing the performance of PhyML 3.0. Syst Biol.

[69] Le SQ, Gascuel O (2008). An improved general amino acid replacement matrix. Mol Biol Evol.

