# Supplementary material for: Apophysomyces variabilis: draft genome sequence and comparison of predictive virulence determinants with other medically important Mucorales
Source: BMC Genomics. 2017 Sep 18;18:736. doi: 10.1186/s12864-017-4136-1 (PMC5604411; doi:10.1186/s12864-017-4136-1)
Supplement: Supplementary file 1 — Gene ontology (GO) of Apophysomyces genomes. Figure S2. Phylogenetic analysis of Apophysomyces species with other Mucorales. (DOC 376 kb) [file 12864_2017_4136_MOESM1_ESM.doc]

**Additional files 2.**

**Figure S1.** **Gene ontology (GO) of *Apophysomyces* genomes.** Different GO functions assigned in *Apophysomyces* genomes a) biological process, b) cellular component, c) molecular function.

**Figure S2. Phylogenetic analysis of *Apophysomyces* species with other *Mucorales***. Phylogenetic tree constructed using 72 single copy genes from 14 fungal species by LG amino acid substitution model with 100 bootstrap replications.


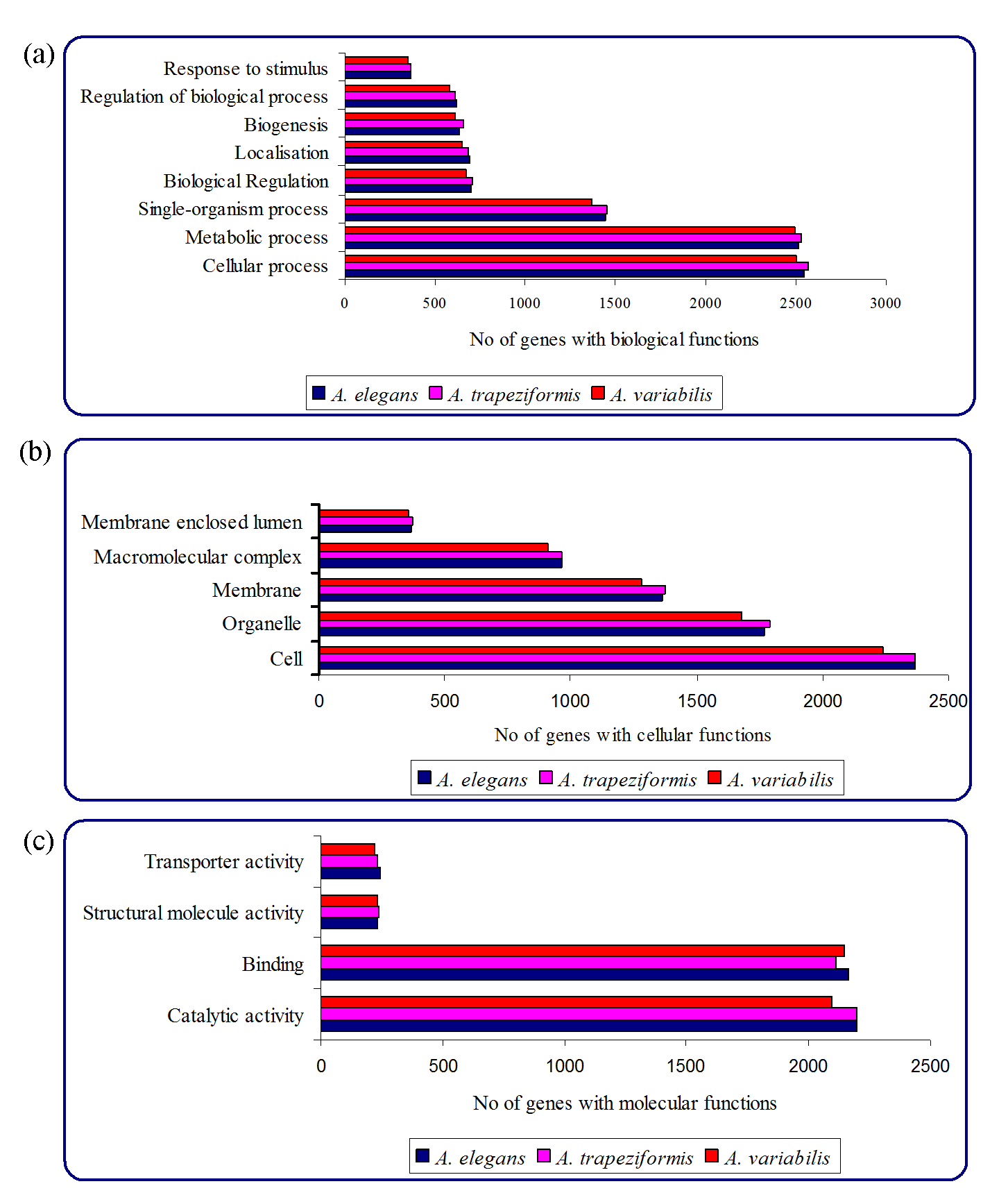


**Figure S1.** **Gene ontology (GO) of *Apophysomyces* genomes.**


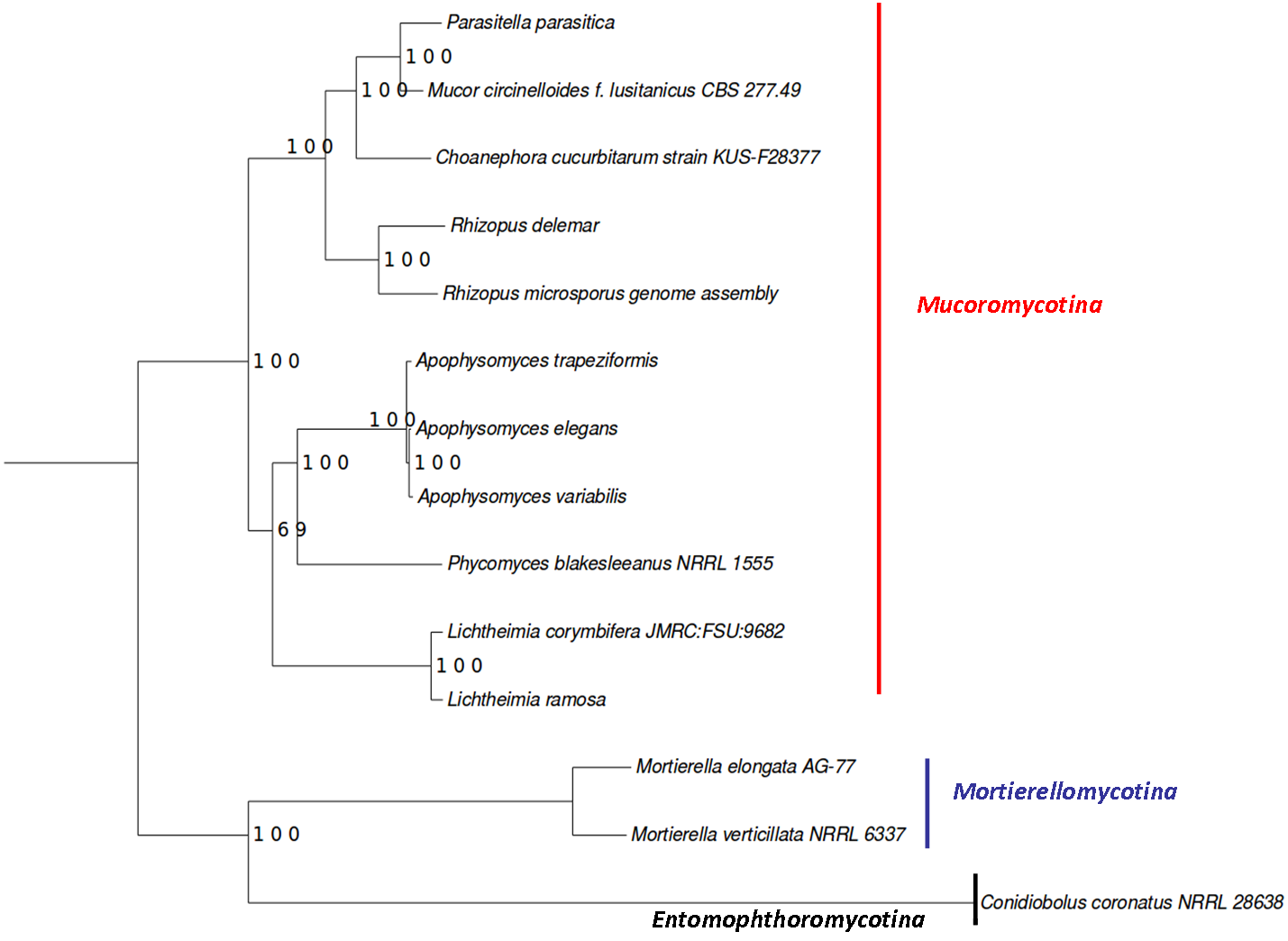


**Figure S2. Phylogenetic analysis of *Apophysomyces* species with other *Mucorales***.
