# Supplementary material for: Apophysomyces variabilis: draft genome sequence and comparison of predictive virulence determinants with other medically important Mucorales
Source: BMC Genomics. 2017 Sep 18;18:736. doi: 10.1186/s12864-017-4136-1 (PMC5604411; doi:10.1186/s12864-017-4136-1)
Supplement: Supplementary file 2 — Non coding RNAs in genomes of three Apophysomyces species. Table S2. Carbohydrate active enzymes (CAZymes) of Apophysomyces species. Table S3. GenBank accession numbers of whole genome sequences used in phylogenetic analysis of Apophysomyces species and orthoMCL analysis. (DOC 163 kb) [file 12864_2017_4136_MOESM2_ESM.doc]

**Additional file-1**

**Table S1.** Non coding RNA copies in genomes of three *Apophysomyces* species

| **Non coding RNAs** | **A. variabilis**  **(N=)** | **A. elegans**  **(N=)** | **A. trapeziformis**  **(N=)** |
| --- | --- | --- | --- |
| Eukaryotic large subunit ribosomal RNA | 25 | 20 | 36 |
| **Bacterial large subunit ribosomal RNA** | 14 | 8 | 28 |
| **Archaeal large subunit ribosomal RNA** | 13 | 7 | 27 |
| Eukaryotic small subunit ribosomal RNA | 7 | 3 | 13 |
| **Bacterial small subunit ribosomal RNA** | 5 | - | 10 |
| **Archaeal small subunit ribosomal RNA** | 6 | 3 | 11 |
| Microsporidia small subunit ribosomal RNA | 7 | 3 | 13 |
| 5S ribosomal RNA (5S rRNA) | 2 | 1 | 4 |
| 5.8 S ribosomal RNA (5.8S rRNA) | 2 | 3 | 5 |
| 5’ ureB sRNA | 1 | 1 | 1 |
| Histone 3' UTR stem-loop | 1 | - |  |
| Group I catalytic intron | 2 | - | 2 |
| Metazoan signal recognition particle RNA | 1 | - | 1 |
| microRNA mir-689 | 1 | - | - |
| **microRNA mir-233** | - | 1 | - |
| Pseudoknot of the domain G(G12) of 23S ribosomal RNA | 1 | - | - |
| Protozoan signal recognition particle RNA | 1 | 1 | 1 |
| RNase MRP | 1 | 1 | 1 |
| Nuclear RNase P | 1 | 1 | 1 |
| small nucleolar RNA snR35 | 1 | 1 | 1 |
| Small nucleolar RNA SNORA53 | 1 | 1 | 1 |
| Small nucleolar RNA Z195/SNORD33/SNORD32 family | 1 | 1 | 1 |
| **Small nucleolar RNA snR75** | 1 | 1 | 1 |
| Small nucleolar RNA snR60/Z15/Z230/Z193/J17 | 2 | 2 | 2 |
| Small nucleolar RNA snR7 | 1 | - | - |
| TPP riboswitch (THI element) | 2 | 2 | 2 |
| U1 spliceosomal RNA | 2 | 2 | 2 |
| U2 spliceosomal RNA | 3 | 3 | 4 |
| Small nucleolar RNA U3 | 2 | 4 | 2 |
| U4 spliceosomal RNA | 1 | 1 | 1 |
| U5 spliceosomal RNA | 2 | 2 | 1 |
| U6 spliceosomal RNA | 3 | 2 | 2 |
| U6atac minor spliceosomal RNA | 1 | 1 | 1 |
| U11 spliceosomal RNA | 1 | 1 | 1 |

Note: ‘N’ denotes number of non coding RNAs as predicted by Infernal tool.

**Table S2.** Carbohydrate active enzymes (CAZymes) of *Apophysomyces* species.

| **Carbohydrate active enzymes** | ***A. variabilis*** | ***A. elegans*** | ***A. trapeziformis*** |
| --- | --- | --- | --- |
| **Glycoside hydrolase (GHs)**  **(N=)** | **97** | **92** | **95** |
| GH109 | 5 | 3 | 4 |
| GH125 | 2 | 2 | 4 |
| GH15 | 2 | 3 | 2 |
| GH16 | 5 | 5 | 6 |
| GH17 | 2 | 3 | 2 |
| GH18 | 13 | 13 | 12 |
| GH20 | 4 | 4 | 3 |
| GH24 | 2 | 2 | 2 |
| GH25 | 8 | 2 | 4 |
| GH27 | 4 | 3 | 2 |
| GH29 | 0 | 1 | 3 |
| GH3 | 5 | 5 | 5 |
| GH31 | 4 | 4 | 4 |
| GH35 | 1 | 1 | 1 |
| GH36 | 3 | 2 | 3 |
| GH37 | 5 | 5 | 5 |
| GH38 | 2 | 2 | 2 |
| GH46 | 3 | 3 | 3 |
| GH47 | 6 | 6 | 6 |
| GH5 | 6 | 6 | 6 |
| GH63 | 1 | 1 | 1 |
| GH72 | 2 | 3 | 2 |
| GH73 | 2 | 3 | 3 |
| GH76 | 1 | 1 | 1 |
| GH8 | 1 | 1 | 1 |
| GH81 | 1 | 1 | 1 |
| GH85 | 2 | 2 | 2 |
| GH9 | 4 | 4 | 4 |
| GH92 | 1 | 1 | 1 |
| **Glycosyl transferase (GTs) (N=)** | **118** | **113** | **125** |
| GT1 | 12 | 10 | 14 |
| GT2 | 21 | 20 | 22 |
| GT4 | 6 | 7 | 6 |
| GT5 | 2 | 2 | 2 |
| GT8 | 3 | 3 | 3 |
| GT10 | 1 | 1 | 1 |
| GT11 | 1 | 1 | 1 |
| GT15 | 8 | 9 | 9 |
| GT20 | 5 | 5 | 5 |
| GT21 | 1 | 1 | 1 |
| GT22 | 3 | 3 | 3 |
| GT23 | 1 | 0 | 1 |
| GT24 | 1 | 1 | 1 |
| GT28 | 1 | 1 | 2 |
| GT31 | 3 | 3 | 3 |
| GT32 | 5 | 5 | 6 |
| GT33 | 1 | 1 | 1 |
| GT34 | 2 | 2 | 2 |
| GT37 | 0 | 1 | 1 |
| GT39 | 6 | 5 | 6 |
| GT47 | 1 | 1 | 1 |
| GT48 | 3 | 3 | 3 |
| GT49 | 8 | 7 | 9 |
| GT50 | 1 | 1 | 1 |
| GT57 | 2 | 2 | 2 |
| GT62 | 4 | 3 | 4 |
| GT64 | 2 | 1 | 2 |
| GT66 | 1 | 1 | 0 |
| GT68 | 4 | 4 | 4 |
| GT69 | 1 | 1 | 1 |
| GT71 | 3 | 3 | 3 |
| GT76 | 1 | 1 | 1 |
| GT77 | 4 | 4 | 4 |
| **Carbohydrate esterase (CEs)**  **(N=)** | **75** | **81** | **76** |
| CE1 | 14 | 14 | 14 |
| CE2 | 1 | 1 | 1 |
| CE4 | 19 | 24 | 21 |
| CE6 | 1 | 1 | 1 |
| CE7 | 1 | 1 | 2 |
| CE9 | 1 | 1 | 2 |
| CE10 | 29 | 31 | 27 |
| CE12 | 1 | 1 | 1 |
| CE14 | 1 | 1 | 1 |
| CE15 | 0 | 1 | 0 |
| CE16 | 7 | 5 | 7 |
| **Carbohydrate-binding module (CBMs) (N=)** | **51** | **51** | **54** |
| CBM12 | 2 | 2 | 2 |
| CBM13 | 25 | 24 | 24 |
| CBM14 | 0 | 0 | 2 |
| CBM19 | 3 | 4 | 4 |
| CBM32 | 3 | 2 | 2 |
| CBM43 | 2 | 3 | 2 |
| CBM48 | 1 | 1 | 1 |
| CBM5 | 2 | 2 | 3 |
| CBM50 | 11 | 13 | 12 |
| CBM68 | 2 | 0 | 2 |
| **Polysaccharide lyase (PLs)**  **(N=)** | **3** | **3** | **3** |
| PL8 | 2 | 2 | 2 |
| PL14 | 1 | 1 | 1 |
| **Total (N=)** | **344** | **340** | **353** |

Note: ‘N’ denotes number of CAZymes as predicted by dbCAN database.

**Table S3.** GenBank accession numbers of whole genome sequences used in phylogenetic analysis of *Apophysomyces* species and orthoMCL analysis.

| **S. No** | **Species** | **Strain IDs** | **NCBI genome (GenBank) accession numbers** |
| --- | --- | --- | --- |
| 1 | *Apophysomyces elegans** | B7760 | JNDQ00000000.1 |
| 2 | *Apophysomyces trapeziformis** | B9324 | JNDP00000000.1 |
| 3 | *Apophysomyces variabilis** | NCCPF 102052 | MZZL00000000 |
| 4 | *Aspergillus fumigatus* | Af293 | AAHF00000000.1 |
| 5 | *Botryotinia fuckeliana B05.10* | B05.10 | AAID00000000.2 |
| 6 | *Choanephora cucurbitarum* | KUS-F28377 | LUGH00000000.1 |
| 7 | *Conidiobolus coronatus* | NRRL 28638 | JXYT00000000.1 |
| 8 | *Coprinopsis cinerea* | Okayama7-130 | AACS00000000.2 |
| 9 | *Cryptococcus neoformans var. grubii* | H99 | GCA_000149245.3 |
| 10 | *Kluyveromyces marxianus* | DMKU3-1042 | GCA_001417885.1 |
| 11 | *Lichtheimia corymbifera** | JMRC:FSU:9682 | CBTN000000000.1 |
| 12 | *Lichtheimia ramosa* | B5792 | JNEP00000000.1 |
| 13 | *Magnaporthe oryzae* | 70-15 | GCA_000002495.2 |
| 14 | *Monosiga brevicollis MX1* | MX1 | GCA_000002865.1 |
| 15 | *Mortierella elongata* | AG-77 | LYLZ00000000.1 |
| 16 | *Mortierella verticillata* | NRRL 6337 | AEVJ00000000.1 |
| 17 | *Mucor circinelloides f. lusitanicus* | CBS 277.49 | AMYB00000000.1 |
| 18 | *Neurospora crassa* | OR74A | AABX00000000.3 |
| 19 | *Parasitella parasitica* | CBS 412.66 | CCXP00000000.1 |
| 20 | *Phycomyces blakesleeanus* | NRRL 1555(-) | AMYC00000000.1 |
| 21 | *Rhizopus microsporus* | RMATCC62417 | CCYT00000000.1 |
| 22 | *Rhizopus arrhizus** | RA 99-880 | AACW00000000.2 |
| 23 | *Rhizoctonia solani* | AG-3 | JATN00000000.1 |
| 24 | *Rhizomucor miehei** | CAU432 | AGBC00000000.1 |
| 25 | *Saccharomyces cerevisiae* | S288c | GCA_000146045.2 |
| 26 | *Schizophyllum commune* | H4-8 | ADMJ00000000.1 |
| 27 | *Scleroderma citrinum* | Foug-A | JMDU00000000.1 |
| 28 | *Serpula lacrymans var. lacrymans* | S7.9 | AEQB00000000.1 |
| 29 | *Sporothrix schenckii 1099-18* | 1099-18 | AXCR00000000.1 |
| 30 | *Trichosporon asahii var. asahii* | CBS 2479 | ALBS00000000.1 |

* Isolates used in orthoMCL analysis.
